# Dynamically switchable magnetic resonance imaging contrast agents

**DOI:** 10.1002/EXP.20210009

**Published:** 2021-09-30

**Authors:** Qiyue Wang, Zeyu Liang, Fangyuan Li, Jiyoung Lee, Liang Ee Low, Daishun Ling

**Affiliations:** ^1^ Institute of Pharmaceutics, College of Pharmaceutical Sciences Zhejiang University Hangzhou Zhejiang P. R. China; ^2^ Hangzhou Institute of Innovative Medicine, College of Pharmaceutical Sciences Zhejiang University Hangzhou Zhejiang P. R. China; ^3^ Department of Biomedical‐Chemical Engineering The Catholic University of Korea Gyeonggi‐do Republic of Korea; ^4^ Biofunctional Molecule Exploratory (BMEX) Research Group, School of Pharmacy Monash University Malaysia Selangor Darul Ehsan Malaysia; ^5^ National Center for Translational Medicine, Frontiers Science Center for Transformative Molecules, School of Chemistry and Chemical Engineering Shanghai Jiao Tong University Shanghai P. R. China; ^6^ Key Laboratory of Biomedical Engineering of the Ministry of Education, College of Biomedical Engineering & Instrument Science Zhejiang University Hangzhou Zhejiang P. R. China

**Keywords:** dynamic MRI contrast agents, medical diagnosis, microenvironmental stimuli

## Abstract

Contrast agents can improve the sensitivity and resolution of magnetic resonance imaging (MRI) by accelerating the relaxation times of surrounding water protons. The MRI performances of contrast agents are closely related to their structural characteristics, including size, shape, surface modification, and so on. Recently, dynamically switchable MRI contrast agents that can undergo structural changes and imaging functional activations upon reaching the disease microenvironment have been developed for high performance MRI. This perspective highlights the ingenious design, controllable structural transformation, and tunable imaging property of dynamic MRI contrast agents. Additionally, the current challenges of the dynamic MRI contrast agents for medical diagnosis are discussed. Furthermore, the future integration of high‐resolution ultra‐high field MRI technology and cutting‐edge dynamic MRI contrast agents for non‐invasive histopathological level accurate detection of microscopic lesions are commented.

## Graphical abstract

This perspective highlights the recent development of dynamically switchable MRI contrast agents that can achieve structural transformation and controllable MR signal activation at the disease site in response to various pathological stimuli. Moreover, current challenges and future perspectives of dynamic contrast agents for the accurate detection of microscopic lesions are commented.

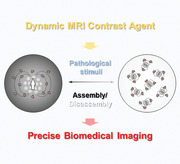

## 1 INTRODUCTION

Magnetic resonance imaging (MRI), as one of the most important imaging modalities in the clinic is a non‐invasive imaging technique characterized by high spatial resolution.^[^ ^1^ ^]^ Nevertheless, the poor sensitivity of MRI remains the impediment for the accurate detection of disease lesions or anatomical changes. Therefore, MRI contrast agents that can shorten the longitudinal (T1) or transverse (T2) relaxation times of water protons in the region of interest are widely used to differentiate the biological targets from normal tissue.^[^ ^2^ ^]^ Based on their effects on relaxation processes of water protons, the contrast agents can be classified into T1 contrast agents with positive contrast enhancement and T2 contrast agents with negative contrast enhancement.^[^ ^3^ ^]^ The MRI signals of the conventional contrast agents are linearly dependent on their local concentrations, thus the pharmacokinetics and biodistributions of contrast agents play vital roles in their biomedical imaging performance.^[^ ^3^ ^]^ As a result, the strategy to improve the disease targeting capability of contrast agents is critical for the enhanced MRI performance.^[^ ^4^ ^]^ Moreover, since the imaging performance of MRI contrast agent also relies on its structural features, including size, shape, chemical composition, surface modification, and so on, enormous efforts have been devoted to improve the MRI performance by regulating the structures of contrast agents.^[^ ^1^, ^5^ ^]^

Despite significant progress in the improvement of the contrasting effects of MRI contrast agents, the conventional local concentration‐dependent contrast agent‐enhanced MRI approach remains difficult to distinguish tiny lesions from the surrounding normal tissues, due to the multiple in vivo physiological barriers that hinder the precisely targeted delivery.^[^ ^6^ ^]^ In recent decades, the development of stimuli‐responsive biomaterials inspired researchers to exploit disease microenvironment‐tailored MRI contrast agents.^[^ ^7^ ^]^ Owing to the enhanced T2 relaxivity of the assembled magnetic nanoparticles compared with the dispersed counterparts, the stimuli‐responsive self‐assembling nanomaterials enable the specific amplification of T2 MRI signal at the disease site.^[^ ^1^, ^8^ ^]^ For example, Long et al. synthesized two sets of tumor‐targeted iron oxide nanoparticles (IONP) that could respond to the overexpressed matrix metalloproteinase enzyme in tumors and give rise to the copper‐free click conjugation among IONP, thereby leading to the formation of IONP assembly with T2 signal enhancement properties at the tumor sites.^[^ ^8b^ ^]^ Nevertheless, these microenvironment‐tailored self‐assembling nanomaterials have limited functionality to improve imaging sensitivity. Also, the responsive T2 signal enhancement could be easily confused with intrinsic hypointense areas in the body.^[^ ^9^ ^]^

In contrast, a dynamically switchable MRI contrast agent, which can realize controllable “turn on/off” adjustment of imaging signal in disease site and normal tissue, holds promising potential to solve the aforementioned problems. Surface ligands play an essential role in the fabrication and subsequent medical application of dynamic MRI contrast agents. Basically, they endow contrast agents with colloidal stability, biocompatibility, stimuli‐responsivity, and target specificity in harsh biological environments. Among the variety of surface ligands, stimuli‐responsive ligands, including polymers,^[^ ^10^ ^]^ biomacromolecules,^[^ ^11^ ^]^ and small molecules,^[^ ^12^ ^]^ have been exploited to manipulate the smart MRI contrast agents based on their stimuli‐responsive structural and property changes. Moreover, active targeting ligands^[^ ^13^ ^]^ (e.g., angiopep‐2 peptide) and biocompatible polymers^[^ ^14^ ^]^ (e.g., polyethylene glycol (PEG)) can be used to further improve the in vivo targeting efficiency and biosafety of dynamic MRI contrast agents.

**FIGURE 1:**
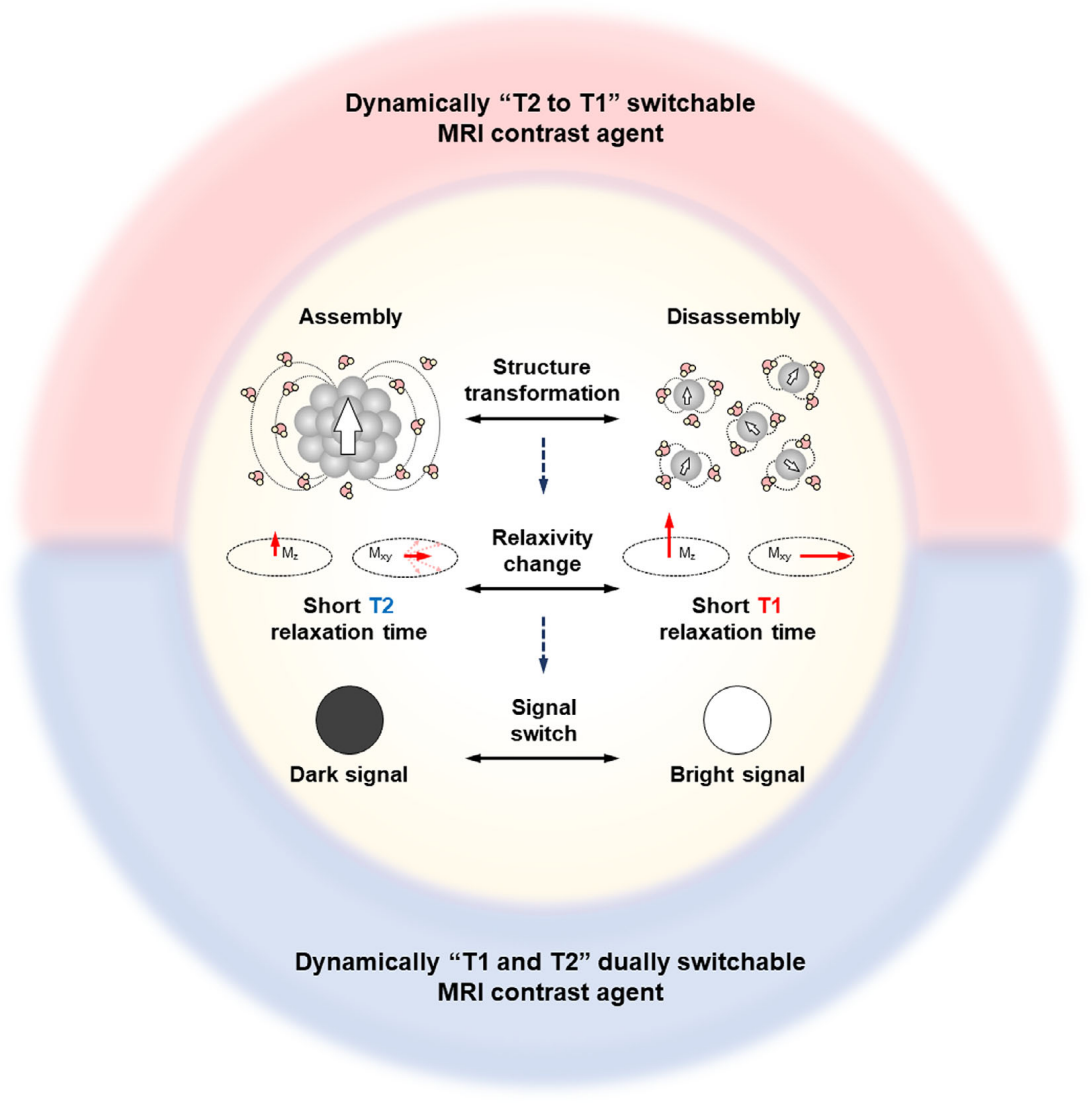
Schematic illustration of dynamically switchable MRI contrast agents that can undergo structural changes upon pathological stimuli, resulting in the MRI signal switch at the disease sites

Upon reaching the disease site, the dynamic MRI contrast agent would undergo structural changes in response to stimuli in the diseased microenvironment, and thus alter its effect on the relaxation of water protons, resulting in the activatable and switchable T1 and/or T2 MRI signals (Figure 1).^[^ ^15^ ^]^ Some typical examples of such dynamically switchable MRI contrast agents are summarized in Table 1. This stimuli‐responsive signal switch strategy shows great promise to improve imaging specificity and realize accurate diagnosis of diseases even at their early stage. In this perspective, the design and fabrication of dynamically switchable MRI contrast agents for precisely regulating the diagnostic functions in refractory diseases are discussed, as well as their current challenges and future developments are commented.

**TABLE 1:** Typical examples of dynamically switchable MRI contrast agents

				r1 (mM^−1^·s^−1^)		r2 (mM^−1^·s^−1^)				
MRI mode	MRI contrast component	Ligand	Stimuli	Before stimulation	After stimulation	Before stimulation	After stimulation	Achievements	Magnetic field (T)	Ref.
“T2 to T1” switch	USION	i‐motif DNA	pH	5.21	4.31	330.32	30.67	Sensitive diagnosis of small HCC	3	[11]
	USION	Hydrazone bond‐based small‐molecular linker ligand	pH	3.2	5.1	108	21.3	Nonlinear MRI signal amplification for tumor diagnosis	3	[12]
	Ultrasmall superparamagnetic iron oxide dots	PEG‐PLA‐ferrocene, angiopep2‐PEG‐PLA, PEG‐PLA‐[Gd^3+^‐DTPA]	Electric field	5.9	9.2	–	–	Visualization of epileptic foci	7	[13]
	USION	PEG‐p(API‐Asp)‐p(DOPA‐Asp)‐Ce6, PEG‐p(API‐Asp)‐p(DOPA‐Asp)‐Ce6	pH	3.30	3.87	43.95	22.45	Early‐stage diagnosis and treatment of tumors	1.5	[10]
“T1 and T2” dual‐switch	SPIO, Mn^2+^ chelate	Disulfide crosslinked micelle	GSH	1.32	3.61	13.19	67.8	Early detection of small intracranial brain tumors	7	[18]
	Organosilica coated IONP, manganese dioxide protrusions	Polyethylene glycol amine	GSH	0.778	8.8	134	244	Imaging‐guided tumor pyroptosis	9	[14]

## 2 DYNAMICALLY “T2 TO T1” SWITCHABLE MRI CONTRAST AGENT

**FIGURE 2:**
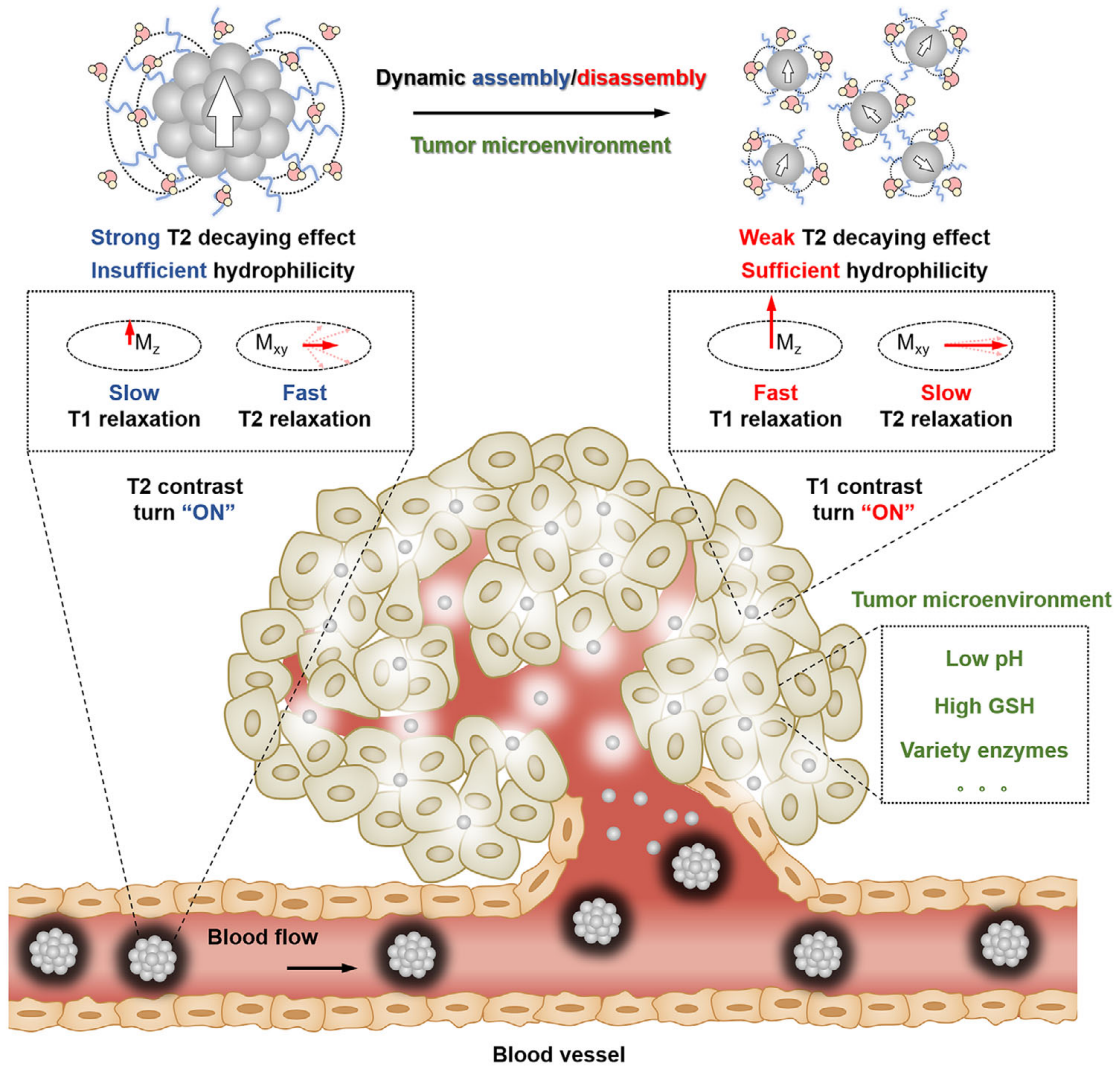
Schematic illustration of the dynamic MRI contrast agent with stimuli‐responsive assembly/disassembly ability to achieve “T2 to T1” contrast switch at the tumor sites

The controlled dynamic assembly of magnetic nanomaterials can be used to achieve “T2 to T1” contrast switch in vivo. Taking tumor as an example, once triggered by the tumor microenvironment, the dynamic MRI contrast agent would change its structure from the assembly state to the dispersed state, accompanied by the enhanced hydrophilicity and attenuated T2 decaying effect, thus exerting “dark” T2 contrast in the normal tissue and “bright” T1 contrast at the disease site (Figure 2). This inverse contrast enhancement between disease site and normal tissue is a promising strategy for highly sensitive and specific detection of lesions.

**FIGURE 3:**
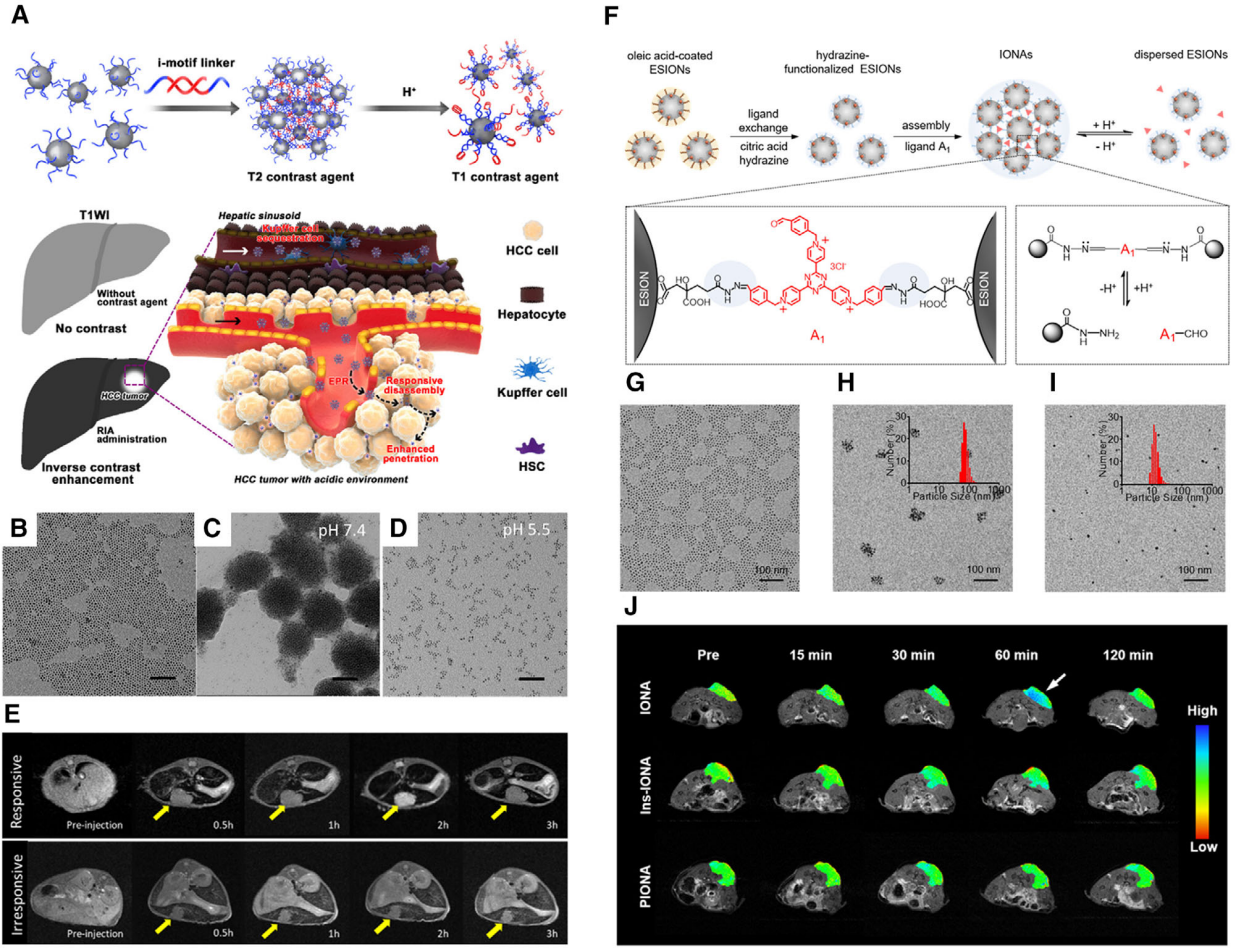
Dynamically “T2 to T1” switchable MRI contrast agents. (A) Schematic illustration of i‐motif DNA‐based iron oxide nanocluster assemblies (RIA) for the diagnosis of HCC. TEM images of (B) ultrasmall iron oxide nanoclusters (USION), (C) RIA at pH 7.4, (D) RIA at pH 5.5 (scale bar = 50 nm). (E) T1‐weighted MRI of RIA and irresponsive assemblies in orthotopic HCC mice. Reproduced with permission.^[^ ^11^ ^]^ Copyright 2018, American Chemical Society. (F) Schematic illustration of small‐molecular aldehyde derivative ligand‐mediated iron oxide nanoparticle assembly (IONA) and its pH‐triggered disassembly. TEM images of (G) USION, (H) IONA at pH 7.4, (I) IONA at pH 5.5. (J) T1‐weighted MRI of tumor‐bearing mice after administration of IONA, pH‐insensitive IONA, and pH‐sensitive polymer‐assisted IONA. Reproduced with permission.^[^ ^12^ ^]^ Copyright 2019, American Chemical Society

Accordingly, based on the acidic tumor microenvironment,^[^ ^16^ ^]^ our group developed a dynamically T2‐T1 switchable MRI contrast agent by cross‐linking the ultrasmall iron oxide nanoclusters (USION) with i‐motif DNA‐based pH‐responsive ligands for the diagnosis of small hepatocellular carcinoma (HCC) (Figure 3A).^[^ ^11^ ^]^ Upon reaching the acidic HCC site, the iron oxide nanocluster assemblies (RIA) disassembled into dispersed USION due to the pH‐responsive structural change of the i‐motif DNA sequence (Figure 3B–D). This promoted transformation of the RIA from a T2 contrast agent (with r_2_/r_1_ of ∼63) to T1 contrast agent (with r_2_/r_1_ of ∼7). As a result, the RIA could simultaneously darken normal liver and brighten HCC under T1‐weighted MRI, significantly enhancing the imaging contrast between normal liver and HCC. As compared with the single‐phase contrast‐enhanced clinical liver‐specific contrast agent, the RIA‐based inverse contrast enhancement strategy can clearly distinguish between normal liver and HCC tissues to realize the highly sensitive detection of early‐stage small HCC (<1 cm in size), which is critical for timely curative treatments (Figure 3E). In addition, we further fabricated a pH‐responsive IONP assembly (IONA) mediated by the small‐molecular ligands composed of hydrazone bonds (Figure 3F–I).^[^ ^12^ ^]^ The cleavage of hydrazone bonds under the acidic tumor microenvironment induces disassembly of the IONA, which leads to specific amplification of T1 MR signals in the tumor site (Figure 3J).

Besides tumors, researchers have also developed “T2 to T1” switchable MRI contrast agent for other refractory diseases. The abnormal electrical activity is a unique characteristic of epilepsy,^[^ ^17^ ^]^ which could be used as the trigger for designing dynamic MRI contrast agent. Recently, Wang et al. developed an electro‐responsive magnetic micelle via assembly of ultrasmall superparamagnetic iron oxide (SPIO) dots using three poly(ethylene oxide)‐poly‐(lactic acid) (PEG‐PLA) derivatives: PEG‐PLA‐ferrocene with electric responsiveness, PEG‐PLA‐[Gd^3+^‐diethylenetriamine penta‐acetic acid (DTPA)] with paramagnetism and angiopep2‐PEG‐PLA with brain‐targetability.^[^ ^13^ ^]^ Based on the reversible oxidation of ferrocene under electric stimulation, the hybrid micelle‐based dynamic MRI contrast agent underwent the dissociation of assembly structure in response to abnormal electrical activity during seizures, which increased the distance between the superparamagnetic core and paramagnetic coating, thereby improving the longitudinal relaxivity of PEG‐PLA‐Gd^3+^‐DTPA and thus achieving specific T1 MR signal activation in epileptic foci. The electric‐field‐responsive MRI contrast agent enables the detection of epileptic foci without structural abnormality, which is a prerequisite for surgical intervention of drug‐resistant focal epilepsy. However, the irreversible structural change of the dynamic contrast agent upon electric stimulation may affect the accuracy of determining the actual location of epileptic foci.

## 3 DYNAMICALLY “T1 AND T2” DUALLY SWITCHABLE MRI CONTRAST AGENT

**FIGURE 4:**
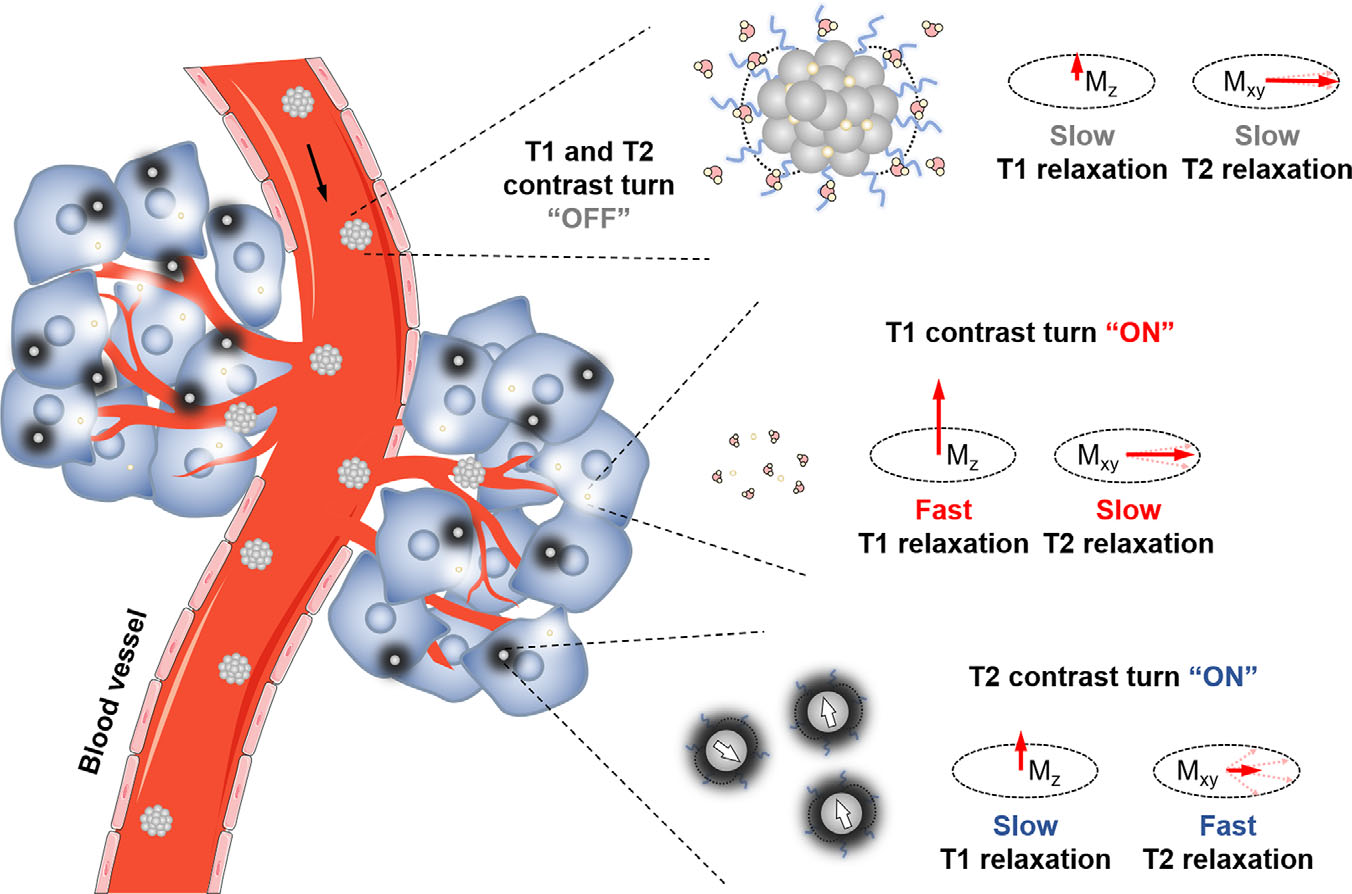
Schematic illustration of the dynamically “T1 and T2” dually switchable MRI contrast agent, which can simultaneously “turn on” T1 and T2 MRI signal when they disassemble and release T1 and T2 contrast materials under pathological stimuli

By manipulating the aggregation state of nanomaterials, the dynamically “T1 and T2” dually switchable MRI contrast agent can be obtained, which exhibits T1 and T2 dual‐quenching effects in the normal tissue, while reveals dually activatable T1 and T2 MR signals at the disease sites.^[^ ^18^ ^]^ As illustrated in Figure 4, the dually activatable contrast agent is typically composed of paramagnetic T1 contrast agents and superparamagnetic T2 contrast agents. Due to the insufficient hydrophilicity as well as magnetic dipole interaction between T1 and T2 contrast materials, the dipole fields from the dually activatable contrast agent are suppressed in the normal tissue, which can induce the quenched T1 and T2 relaxation rates. On interaction with pathological stimuli, the assembly structure of the dually activatable contrast agent is destroyed, thus leading to an increased distance between T1 and T2 contrast materials, resulting in the T1 and T2 signal recovery (Figure 4).

**FIGURE 5:**
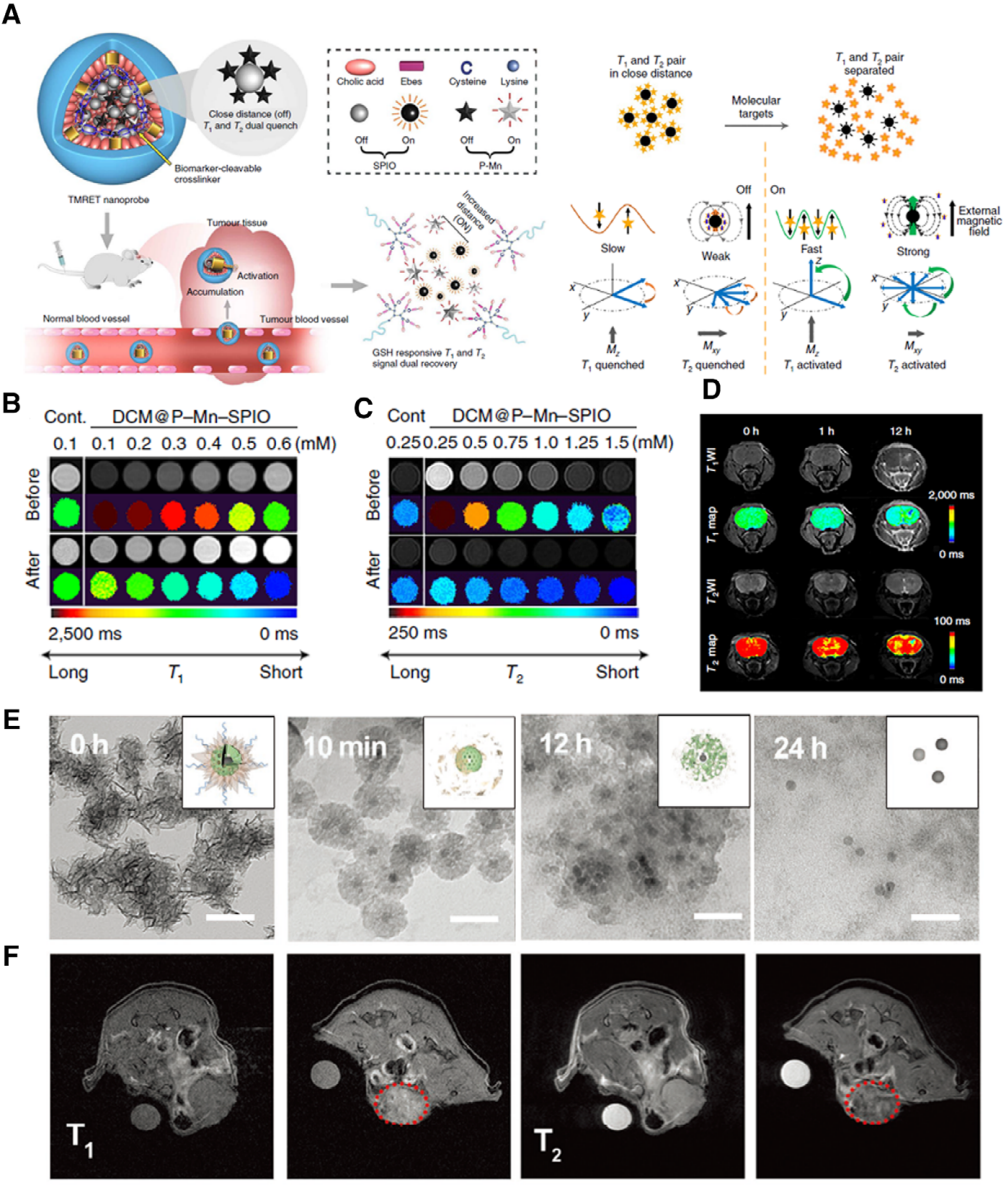
Dynamically “T1 and T2” dually switchable MRI contrast agent. (A) Schematic illustration of the GSH‐responsive disassembly of dually switchable MRI contrast agent (DCM@P‐Mn‐SPIO) and its mechanism for T1 and T2 quenching and recovery. (B) T1‐ and (C) T2‐weighted images of DCM@P‐Mn‐SPIO before and after incubation with GSH. (D) T1‐ and T2‐weighted images and the corresponding mapping images of mice bearing orthotopic brain tumors treated with DCM@P‐Mn‐SPIO. Reproduced with permission.^[^ ^18^ ^]^ Copyright 2020, Springer Nature. (E) TEM images of virus‐spike tumor‐activatable pyroptotic agent (VTPA) after incubation in solutions with 10 mM GSH for different times. (F) T1‐ and T2‐weighted MRI of tumor‐bearing mice before and after 6 h of intravenously injected with VTPA (red dashed circles). Reproduced with permission.^[^ ^14^ ^]^ Copyright 2021, Wiley‐VCH

The glutathione (GSH) concentration in the cytoplasm of tumor cells is at least 4‐folds higher than that of normal cells.^[^ ^19^ ^]^ Such a significant difference can serve as an excellent stimulus to trigger the structural changes of dynamic MRI contrast agent in tumor cells. Recently, Wang et al. fabricated a GSH‐responsive dually switchable MRI contrast agent via encapsulation of SPIO nanoparticles and Mn^2+^ chelates into a disulfide crosslinked micelle (Figure 5A).^[^ ^18^ ^]^ The integrity of the assemblies would be disrupted in response to GSH addition. This induced the increased distance between Mn^2+^ chelate and SPIO, thereby obtaining the dually enhanced T1 and T2 MR signals (Figure 5B,C). This dynamic MRI platform has significantly improved the tumor‐to‐normal tissue contrast ratio and has been successfully applied to detect early‐stage small intracranial brain tumors (Figure 5D).

Recent advances in dynamic contrast agents promote the development of MRI‐guided therapy. For example, our group designed a virus‐spike tumor‐activatable pyroptotic agent (VTPA) for dually switchable MRI‐guided cancer therapy, where organosilica coated IONP was synthesized as the core, which was further surface decorated with spiky manganese dioxide protrusions.^[^ ^14^ ^]^ The spiky structure of VTPA can induce intracellular lysosomal rupture, and respond to tumor intracellular GSH to facilitate the effective release of Mn^2+^ and IONP, eventually leading to the T1‐T2 dual MRI contrast enhancement and synergetic cancer pyroptosis (Figure 5E,F).

Overall, the construction of “T2 and T1” dually switchable contrast agent represents a promising way to achieve accurate disease imaging and diagnosis through double‐checking of the T1 and T2 MRI signals. However, the currently developed dually activatable contrast agents are mainly used for tumor imaging. Thus, it is necessary to further investigate the applications of these smart contrast agents in other refractory diseases, such as neurological disorders, cardiovascular diseases, and so on.

## 4 FUTURE PERSPECTIVE

**FIGURE 6:**
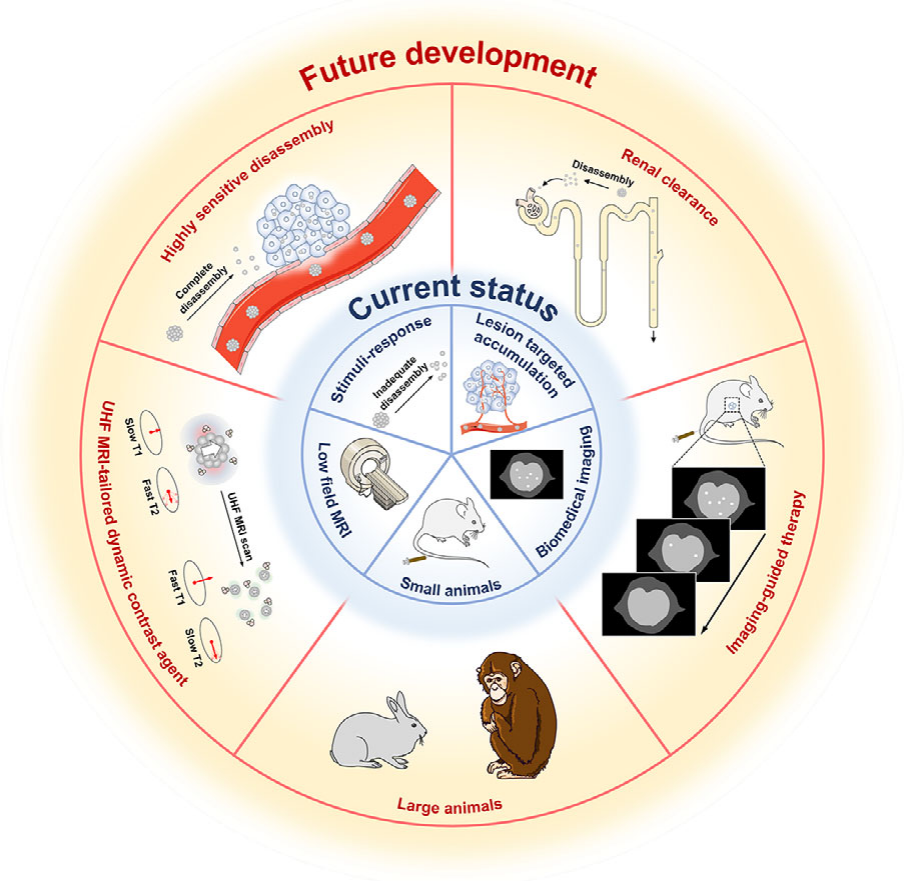
Schematic illustration of the current status and future development of dynamically switchable MRI contrast agents

In recent years, according to the structure–relaxivity relationship of contrast agents, the state‐of‐the‐art dynamic MRI contrast agents have been developed for sensitive detection of refractory diseases. However, the smart dynamic MRI contrast agents are still in the proof‐of‐concept stage and several challenges remained to be addressed before further clinical application (Figure 6). First, oftentimes, there is only a slight physicochemical discrepancy between disease lesions and surrounding normal tissues, which puts forward high requirements on the sensitivity and selectivity of dynamic contrast agents (Figure 6). Since the undesirable nanoparticle aggregation would lead to the false amplification of the T2 MRI signal, the contrast agents should maintain their colloidal stability in vivo under both the assembled or disassembled state. The stimuli‐responsive ligands that interact directly with biological systems play an important role in the colloidal stability and responsiveness of dynamic contrast agents. Thus, the optimization of stimuli‐responsive ligands for dynamic contrast agents, including composition, structure, response range, response time, response accuracy, and specificity, is highly desirable to detect small changes in the disease microenvironment to promote sensitive MRI activation at the disease lesions. Second, despite immense efforts on the chemical design, the lack of systematic toxicological assessment of these smart contrast agents ultimately hinder their clinical translation. On the one hand, the development of renal‐clearable or rapidly biodegradable dynamic contrast agents is helpful to ensure biosafety and biocompatibility; on the other hand, reproducible synthetic methods should be exploited to guarantee the imaging performance of dynamic contrast agents after the large‐scale preparation. Third, it is worth noting that most of the current studies only evaluate the imaging performance of dynamic contrast agents in small animals. In future, it is necessary to verify the imaging performance of dynamic contrast agents by directly comparing their performance with standard‐of‐care diagnostic regimens in large animals, which would provide a more reliable basis for the clinical translation (Figure 6).

In addition, the ultimate goal of biomedical imaging is to provide guidance for further disease treatment. The drug‐loaded dynamic contrast agent is expected to achieve controllable imaging and therapeutic functions for theranostic applications (Figure 6). Furthermore, dynamic contrast agent‐enhanced MRI shows great potential as a novel platform to perform a pre‐surgical assessment, monitor therapeutic response, and detect the residual lesions after surgery.

Moreover, the recently developed ultra‐high field (UHF, ≥7T) MRI is a powerful imaging tool with much higher resolution than conventional MRI.^[^ ^20^ ^]^ Nevertheless, the T1 relaxation times of water protons become much longer in UHF MRI,^[^ ^21^ ^]^ which would decrease the T1 relaxivity of conventional contrast agents at UHF. To develop the UHF‐tailored dynamic MRI contrast agent, a comprehensive understanding of the structure–relaxivity relationship of contrast agent at UHF is urgently needed. The future integration of UHF MRI technology and UHF‐tailored dynamically switchable MRI contrast agents is expected to reach extremely high level imaging performances for the accurate diagnosis of diverse diseases (Figure 6). We anticipate the cutting‐edge dynamically switchable MRI contrast agents, with supreme sensitivity even at UHF, shall eventually achieve the non‐invasive histopathological level visualization of previously undetectable biological entities that are critical to medical diagnosis and therapy.

## CONFLICT OF INTEREST

Daishun Ling is a member of the *Exploration* editorial board. The authors declare no conflict of interest.
